# The Mechanical Properties of Erythrocytes Are Influenced by the Conformational State of Albumin

**DOI:** 10.3390/cells14151139

**Published:** 2025-07-24

**Authors:** Ivana Pajic-Lijakovic, Milan Milivojevic, Gregory Barshtein, Alexander Gural

**Affiliations:** 1 Faculty of Technology and Metallurgy, University of Belgrade, 11000 Belgrade, Serbia; mmilan@tmf.bg.ac.rs; 2 Department of Biochemistry, The Faculty of Medicine, Hebrew University, Jerusalem 91120, Israel; gregoryba@ekmd.huji.ac.il; 3 Blood Bank, Hadassah-Hebrew University Medical Center, Jerusalem 91120, Israel; gural@hadassah.org.il

**Keywords:** unfolding of albumin, extensional flow, discocyte-to-stomatocyte transition, electrostatic interactions, hydrophobic interactions, blood viscoelasticity

## Abstract

The mechanical stability and deformability of erythrocytes are vital for their function as they traverse capillaries, where shear stress can reach up to 10 Pa under physiological conditions. Human serum albumin (HSA) is known to help maintain erythrocyte stability by influencing cell shape, membrane integrity, and resistance to hemolysis. However, the precise mechanisms by which albumin exerts these effects remain debated, with some studies indicating a stabilizing role and others suggesting the opposite. This review highlights that under high shear rates, albumin molecules may undergo unfolding due to normal stress differences. Such structural changes can significantly alter albumin’s interactions with the erythrocyte membrane, thereby affecting cell mechanical stability. We discuss two potential scenarios explaining how albumin influences erythrocyte mechanics under shear stress, considering both the viscoelastic properties of blood and those of the erythrocyte membrane. Based on theoretical analyses and experimental evidence from the literature, we propose that albumin’s effect on erythrocyte mechanical stability depends on (i) the transition between unfolded and folded states of the protein and (ii) the impact of shear stress on the erythrocyte membrane’s ζ-potential. Understanding these factors is essential for elucidating the complex relationship between albumin and erythrocyte mechanics in physiological and pathological conditions.

## 1. Introduction

### 1.1. Albumin

Human serum albumin (HSA), synthesized by the liver, is the most abundant protein in human blood plasma, accounting for 50–60% of total plasma proteins [1]. It is the primary fatty-acid-binding protein in plasma, with seven binding sites for fatty acids that have moderate-to-high affinity [2]. Under normal physiological conditions, albumin has a half-life of about 20 days.

The molecular weight of this globular protein is approximately 66.4 kDa, while its hydrodynamic radius measures about 7 nm in diameter. In human blood, albumin primarily exists as a monomer under normal physiological conditions, with a small fraction (<10%) capable of forming dimers or higher-order oligomers. This aligns with the fact that the isoelectric point (pI) of HSA is approximately 4.7 [3]. Consequently, these molecules are anions at the physiological pH of 7.4. HSA comprises three homologous domains (I, II, and III), each subdivided into two subdomains, which facilitate ligand binding and provide flexibility for transporting various molecules. The physiological concentration of albumin in blood is 40 g/L [1]. HSA is an essential marker of nutritional status and inflammatory response [4,5], as well as the subject’s biological age [6,7]. The physiological functions of albumin include anticoagulant, antioxidant, anti-inflammatory, and anti-platelet aggregation activities [8]. Moreover, some studies report that the HSA concentration is inversely associated with the occurrence of diseases, dysfunction related to inflammation, oxidative stress, and metabolic regulation, as well as mortality [9,10,11,12]. Albumin levels below 3.6 g/dL are linked to higher mortality rates among elderly individuals [13].

Native vs. Modified Albumin: Native human serum albumin (HSA) maintains specific structural domains and binding capacities essential for stabilizing erythrocyte membranes, influencing cell shape, and preventing hemolysis [2]. However, modified forms of albumin—such as oxidized or glycated HSA—exhibit altered structural and functional properties [3,4]. Oxidized albumin can have a reduced binding affinity for fatty acids and other ligands. It may generate reactive oxygen species, potentially increasing oxidative stress on erythrocyte membranes and affecting their deformability [5]. Glycated albumin, often elevated in diabetic patients, may similarly impair albumin’s stabilizing role, contributing to increased erythrocyte aggregation and reduced membrane fluidity [6]. Thus, the functional state of albumin is critical in modulating erythrocyte stability under both physiological and pathological conditions.

Individual Variability: We also acknowledge that personal factors such as age, sex, and inflammatory state could influence albumin–erythrocyte interactions [7]. Aging is associated with increased oxidative modifications of albumin and alterations in plasma protein composition, which may impact albumin’s protective functions [8]. Sex-related hormonal differences might influence oxidative stress levels and albumin modifications [9]. Additionally, inflammatory states can alter albumin levels and promote post-translational modifications (e.g., oxidation and glycation), further impacting erythrocyte stability and membrane interactions [10,11].

### 1.2. Albumin Unfolding

Protein folding remains a key open question in science [14]. Both experimental and theoretical studies, including numerical modeling, have been conducted to investigate this phenomenon. Albumin unfolding can be triggered by mechanical stress, temperature changes, pH shifts, glycation, oxidation, or chemical denaturation [15,16]. Understanding the mechanism behind albumin unfolding is especially important due to the protein’s high biological significance. The processes of albumin folding and unfolding in solution have been studied using various techniques, such as NMR [17,18], ultrasonic methods [19], rheological techniques [20], Raman spectroscopy [21], and circular dichroism [22]. Exposure to certain conditions, like very low (less than 5) or high (greater than 10) pH, heat, freezing temperatures, or high salt concentrations, can cause different protein molecules to unfold, exposing their hydrophobic regions [23].

### 1.3. Serum Albumin Physiological Role

Serum albumin plays several crucial roles in blood: (i) it maintains osmotic pressure; (ii) it transports molecules such as fatty acids, hormones, and cations; (iii) it helps sustain blood pH by acting as a buffer; and (iv) it has antioxidant properties that aid in neutralizing free radicals [3,24]. Additionally, albumin is essential for the antioxidant function of blood plasma against reactive oxygen species [25,26,27].

The influence of albumin on erythrocyte properties: The presence of HSA in plasma significantly affects the mechanical behavior of erythrocytes. Interactions between albumin and the erythrocyte membrane at physiological concentrations are recognized as key factors responsible for the mechanical stabilization of cells [12]. Some studies indicate that HSA enhances the mechanical stability of erythrocytes under shear stress [12,13], while others suggest that erythrocyte stability diminishes due to the presence of HSA [14]. A decrease in the mechanical stability of erythrocytes can be induced by structural changes of the membrane under higher shear rates. This cause-and-effect relationship primarily depends on the magnitude of the shear rate. Under normal blood pH, albumin molecules are negatively charged [15,16] and interact with the similarly negatively charged erythrocyte membranes primarily through electrostatic forces [17,18]. Two sublayers envelop the negatively charged erythrocyte’s membrane. The inner layer is the Stern sublayer, which consists of cations, while the outer layer consists of anions such as albumin. Consequently, albumin contributes to the zeta potential of the erythrocyte membrane [19]. A higher zeta potential indicates more stable cells [20]. Ions within the sublayers are subject to both electrostatic and hydrodynamic forces, which influence their mobility and the thickness of the sublayers. The thickness of these sublayers, along with the zeta potential, decreases with increasing shear stress [21].

Additionally, albumin can induce changes in erythrocyte shape [28,29]. Therefore, supplementing long-stored erythrocytes with 20% albumin or washing them in an albumin-containing (0.2%) solution reversed all degrees of echinocytosis towards discocytosis [22]. As Reinhart et al. [22] demonstrated, albumin has the capacity to reverse echinocytosis induced by RBC storage. Conversely, Jay [14] demonstrated that when healthy erythrocytes are suspended in Ringer’s solution, less than 2% of the cells are cup-shaped. However, adding HSA to the suspension elevates the number of cup-shaped cells to 15–50%. Later, Reinhart and Chien [23] confirmed that the presence of albumin in cell suspension leads to an increase in the concentration of stomatocytes. In other words, the presence of albumin in the cell suspension makes stomatocytes the preferred erythrocyte shape.

Selevan et al. [24] studied the role of albumin in influencing erythrocyte stiffness. In their study, the authors employed two techniques: the passage of cells through porous polycarbonate membranes and the use of optical tweezers. They demonstrated a significant increase in cell stiffness following an increase in albumin concentration.

The interactions between albumin and erythrocyte membranes are highly relevant in several pathological contexts. For example:

Diabetes Mellitus: Chronic hyperglycemia leads to non-enzymatic glycation of proteins, including HSA [25]. Glycated albumin exhibits altered conformation and reduced binding capacity [26], potentially impairing its ability to stabilize erythrocyte membranes. Moreover, diabetes is associated with increased oxidative stress [27], further modifying albumin’s structure and function [30]. These changes could exacerbate erythrocyte deformability deficits [31], contributing to microvascular complications and impaired blood flow [32].

Liver Disease: In liver failure, serum albumin levels are often reduced, and the fraction of structurally modified albumin (e.g., oxidized or nitrosylated) increases [33]. Such modifications diminish albumin’s protective effects on erythrocyte membranes, potentially leading to increased hemolysis or altered rheological properties of blood [5,34]. This may worsen complications like portal hypertension and contribute to microcirculatory dysfunction [35].

More broadly, any pathological condition that alters the concentration, structure, or binding properties of albumin could affect erythrocyte stability and mechanics [36]. Additionally, changes in albumin levels have a significant effect on erythrocyte aggregation [35].

Our review suggests that under conditions of elevated shear stress, unfolded albumin may interact differently with the erythrocyte membrane, potentially disrupting membrane integrity and function.

Understanding these mechanisms could have several clinical implications:

- It may guide the development of biomarkers based on albumin structural modifications to predict erythrocyte-related complications in disease.
- It could inform therapeutic strategies aimed at preserving albumin’s native conformation or supplementing functional albumin in patients with low or dysfunctional albumin levels.
- It may influence decisions regarding transfusion practices or blood storage, particularly in patients at risk for erythrocyte mechanical fragility.

Thus, exploring the interplay between albumin’s structural state and erythrocyte mechanics has significant clinical relevance for diseases where both albumin dysfunction and impaired microcirculation play key roles.

### 1.4. Role of Shear Stress

Blood flow conditions: In vivo, blood flow refers to the movement of blood through vessels from the arteries to the capillaries and veins. The heart pumps the blood through a network of branching blood vessels with gradually decreasing diameters, facilitating microcirculation. The generated level of pressure determines the force that blood exerts on the vessel walls as it is propelled through the vessels. Like all fluids, blood flows from areas of high pressure to areas of low pressure. Circulation and shear flow of viscoelastic fluids, such as blood, in Couette flow can induce instabilities in the form of secondary flow and can even lead to turbulence at lower Reynolds numbers [36]. These instabilities can feed back on the unfolding of albumin. However, albumin remains relatively stable under Poiseuille shear flow within the physiological range of shear rates from 10 s^−1^ in veins to 1000 s^−1^ in capillaries [37]. In biological settings, shear stress in blood vessels typically ranges from 0.1 to 1 Pa. Still, in extreme cases (such as atherosclerosis or turbulence), shear stress is significantly increased and can exceed 10 Pa [38]. Additionally, shear flow also induces the generation of normal stress components. This aligns with the fact that blood is an anisotropic and viscoelastic fluid [39]. The viscoelasticity of blood has been characterized by non-linear upper-convected Maxwell and Jeffreys models, as well as by the generalized Maxwell model and the Oldroyd-B model [40,41]. The primary features of these models include (i) the ability of stress to relax under a constant strain rate and (ii) the relaxation of strain rate under constant stress conditions (applicable solely to the Jeffreys model). The first and second normal stress differences can quantify the anisotropic behavior of blood. The first normal stress difference of blood is positive, primarily due to the deformability of erythrocytes, while the second one is much smaller and nearly equal to zero [42,43]. Positive normal stress difference facilitates extension of blood components in the direction of flow. The corresponding tensional stress is equal to the first normal stress difference, which increases with shear rate [43]. For a shear rate of 500 s^−^^1^, the first normal stress difference of blood is equal to N_1_ = 10 Pa [43]. The generation of tensional stress, as a result of the first normal stress difference, is pronounced for blood osmotic flow through capillaries. The radius of a capillary is about 2–5 µm, while the shear rates in capillaries typically range from 100 s^−^^1^ to 1000 s^−^^1^ [44]. Extensional flow, characterized by the strain rate of ~1000 s^−^^1^, has the potential to unfold thermodynamically and kinetically stable globular proteins [45]. However, no experimental evidence exists for unfolding HSA under these conditions.

Thus, shear stress generated by blood flow reaches tens and hundreds of Pa and can stimulate changes in plasma components and erythrocytes [38]. Under such conditions, albumin molecules can unfold.

### 1.5. Unfolding of Albumin Under Shear Flow

The literature presents conflicting views on the influence of shear flow on the unfolding of albumin. While Jaspe and Hagen [46] have indicated that globular proteins, including albumin, can retain their stable conformation even when subjected to high shear rates, some authors have demonstrated that very high shear stress can initiate protein unfolding [46,47]. Thus, Jaspe and Hagen [46] suggest that “extraordinary shear rates, approximately 107 s^−^^1^, would be required to denature typical small, globular proteins in water”. At the same time, Brückl et al. [48] conclude that shear flow cannot induce the unfolding of rhGH and IgG1 at shear rates of at least 104 s^−1^.

**Figure 1:**
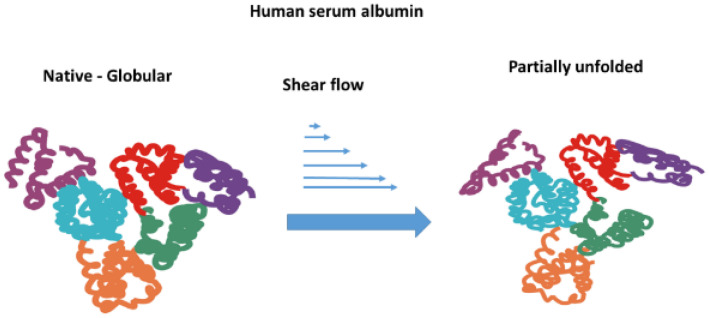
Schematic illustration of albumin unfolding under shear flow that may potentially affect the mechanical stability of erythrocytes.

Specifically, for albumin, Bekard et al. [49] demonstrated that under laminar flow with shear rate of 500 s^−^^1^, it may undergo molecule unfolding. Furthermore, albumin molecules can be unfolded even under very short (0.36–1.8 ms) exposure of diluted bovine serum albumin (BSA) solution to extensional flow [45]. Zocchi [50] studied the unfolding of BSA using atomic force microscopy and noted the stepwise nature of unfolding, with a waiting time of 0.1 s. An increase in osmotic pressure has a feedback impact on the mechanical stability of erythrocytes. Moreover, Kiese et al. demonstrated that unfolding promotes intermolecular interactions and may result in protein aggregation and sub-visible particle formation [51]. Additionally, extensional flow has been shown to induce the unfolding of albumin [45]. Shear stress initiating the process of albumin unfolding is schematically presented in Figure 1.

### 1.6. Shear Stress Sensitivity of Erythrocytes

Shear stress generated during blood flow through blood vessels alters the characteristics of red blood cells. Under the influence of mechanical stress, hemolysis can [52,53,54], together with changes in their morphology (i.e., the transition of red blood cells to stomatocytes) [14], lead to an increase in rigidity [53,55] and an increase in fragility [21]. These changes in cell morphology and stiffness can be related to (i) ATP depletion [56], (ii) phosphatidyl-serine (PS) externalization [57], (iii) a change in protein content within the membrane and the activity of mechanosensitive Piezo1 channels [58,59], (iv) a decrease in the cell surface charge [21], (v) an increase in erythrocyte aggregation [21], and (vi) erythrocyte adhesion to the endothelium [57,60]. Our recent review provides a detailed examination of the impact of mechanical stress on cellular properties and functionality [38].

### 1.7. Factors That Dictate Erythrocyte Mechanical Stability

Mechanical stability of erythrocytes represents their ability to maintain the integrity of the cell membrane under mechanical stress. Less deformable and stiffer cells are prone to mechanical disruption under higher shear rates [61]. The stiffness of the erythrocyte membrane is closely connected with membrane viscoelasticity. The viscoelasticity of the membrane depends on the interplay between (i) the rearrangement of lipids, (ii) the coupling of the bilayer and actin cortex, (iii) the rearrangement of band 3, (iv) the intracellular concentration of calcium, and (v) the flexibility of spectrin filaments [62]. The flexibility of spectrin filaments depends on the persistent length of a given contour. The effective persistent length of spectrin filaments depends on the number of attached band 3 molecules per filament. It follows that band 3 molecules can form low-affinity complexes with spectrin during their lateral diffusion along the membrane [63]. Rearrangement of lipids influences the bilayer bending and consequently impacts the coupling between the bilayer and actin cortex [62,63,64]. The concentration of calcium ions within cells, which is modulated by the function of mechanosensitive Piezo1 channels, plays a crucial role in regulating the viscoelastic properties of the cytoskeleton [65]. The presence of calcium triggers the activation of calpain. This proteolytic enzyme cleaves spectrin and various other cytoskeletal proteins, leading to a reduction in cytoskeletal integrity and an increase in deformability [66,67].

The primary objective of this theoretical analysis is to investigate how albumin may affect the mechanical stability of erythrocytes under higher shear stress, thereby encouraging further research in this field. To develop an accurate constitutive model and describe the viscoelastic properties of the epithelial membrane when albumin is present, additional experiments are required, including (i) measuring the shear rate, shear stress, and the resulting tensile stress in blood flow; (ii) microrheological testing of erythrocyte membrane viscoelasticity, focusing on storage and loss modulus vs. frequencies; (iii) observing shape changes in erythrocytes and the rate of these changes under shear flow; (iv) examining conformational changes in albumin using scattering techniques; and (v) assessing changes in zeta potential. Pajic-Lijakovic and Milivojevic [40] proposed a model of the erythrocyte membrane based on microrheological data from the literature. The next step is to verify this model under the specific conditions discussed in this section.

## 2. Effect of Albumin on Erythrocyte Mechanical Stability

Kamada et al. [68] were the first researchers to analyze the role of plasma components in RBC mechanical trauma. Following his pioneering work, numerous studies have demonstrated that proteins in the medium reduce mechanical stress-related hemolysis [65,66,67,69] and that patients undergoing cardiopulmonary bypass exhibit alterations in erythrocyte morphology, whereas albumin supplementation weakens the observed effect. Moreover, it has been shown that albumin is a protective component of the heat-stable extract of fresh-frozen plasma or warmed plasma supernatant [13,70]. In contrast, ceruloplasmin, γ-globulin, α-2-macroglobulin, and haptoglobin provide only slight protection [13]. Sumpelman and colleagues [71] speculated that this protective effect can be credited to negatively charged proteins that reversibly attach to the cell membrane. Later, other authors suggested that plasma proteins coat the erythrocytes’ surface and help repair damage to the cell membrane caused by mechanical stress [70,72].

It is necessary to note that the results of the experiments presented in the previous paragraph refer to flow conditions where albumin molecules are not unfolded. What will happen when this threshold is crossed, and the cells are subjected to mechanical stress under conditions when the albumin molecules have unfolded?

Below, we examine albumin’s influence on the mechanical stability of erythrocytes under higher shear rates that stimulate the generation of tensional stress, characteristic of the flow of viscoelastic and anisotropic fluids such as blood. To address this issue, we discuss the impact of shear flow on the conformational state of albumin molecules, with a focus on the interactions between albumin and the erythrocyte membrane. Our consideration is based on relevant theoretical analyses and accumulated experimental data.

The experimental evidence of the role of albumin in the mechanical stabilization of erythrocytes is summarized in Table 1:

The impact of albumin on structural changes of erythrocytes can be discussed in the context of two possible scenarios. One scenario is associated with increased osmotic pressure [70], while the other occurs under constant osmotic pressure, thereby maintaining an isotonic condition. An increase in osmotic pressure, caused by albumin folding, can induce a discoid-to-stomatocyte transition. When albumin unfolds, the total number of molecules remains unchanged; however, the thermodynamically active number of hydrophilic residues increases due to several factors: (i) the higher configurational entropy of the unfolded protein chains; (ii) greater interaction with water molecules, which functionally makes the protein “larger” in an osmotic sense; and (iii) enhanced electrostatic repulsion if additional charged groups become exposed, thereby increasing the protein’s osmotic activity. At the same time, the constant osmotic pressure scenario is related to the establishment of two charged sublayers around erythrocytes, which are capable of mechanically stabilizing the cell membranes.

### 2.1. Scenario 1 of Cell Response

As we pointed out earlier, when albumin maintains its globular shape, the interactions between albumin molecules and the erythrocyte membrane are mainly electrostatic [18]. In contrast, as albumin molecules denature, their hydrophobic interactions with the membrane become increasingly important. Albumin does not unfold in a simple two-state manner. It exhibits multiple intermediate conformations, as reported in [27,28]. These transitions may be reversible or irreversible, depending on the duration and intensity of the perturbation. The shift of albumin between its folded and unfolded forms is regulated by non-covalent forces—such as hydrophobic, electrostatic, and hydrogen bonds—as well as covalent disulfide bonds. The interplay of these interactions between unfolded albumin and the erythrocyte membrane influences cell mechanical stability by changing the membrane’s stiffness and the bilayer’s bending modulus. These interactions include hydrophobic effects, electrostatic forces, and lipid binding or sequestration.

Unfolded albumin molecules expose hydrophobic regions previously buried within the protein, altering their interaction with surrounding water molecules and consequently changing the amount of osmotically unresponsive water (OUW) [73]. OUW refers to the fraction of water within cells or protein solutions that does not participate in osmotic activities, often due to strong interactions with macromolecules such as proteins. A decrease in OUW implies that more water molecules are free to engage in osmotic processes, thereby increasing osmotic pressure [74]. An increase in osmotic pressure affects erythrocyte volume and shape, the membrane’s stiffness, the rearrangement of lipids within the bilayer, and the fragility [1,75].

**Figure 2:**
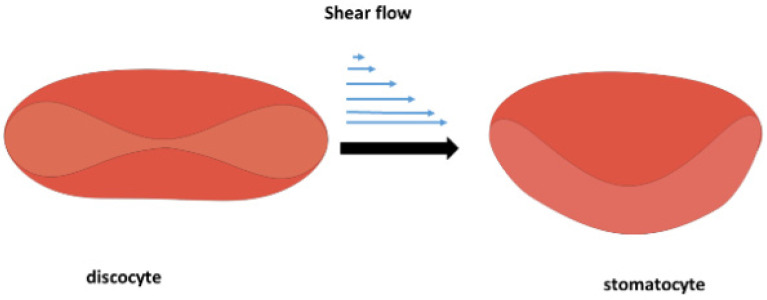
Schematic representation of the discocyte-to-stomatocyte transition under shear flow caused by albumin unfolding. The two factors, (i) an increase in osmotic pressure and (ii) the generation of tensional stress due to blood’s viscoelasticity and anisotropy, contribute to the mechanical instability of erythrocytes, leading to the transition from discocyte to stomatocyte.

At elevated shear rates, when albumin undergoes unfolding, causing an increase in osmotic pressure, the blood tonicity changes from isotonic to hypertonic [76]. It follows that the normal stress difference, responsible for the extension of albumin molecules and their subsequent unfolding, increases with the blood flow shear rate [43]. When erythrocytes are placed in a hypertonic environment, water exits the cells due to osmosis, resulting in a decrease in cellular volume [77]. These two factors—(i) an increase in osmotic pressure and (ii) the generation of tensional stress—contribute to the mechanical instability of erythrocytes, leading to the discocyte-to-stomatocyte transition. The discocyte-to-stomatocyte transition under shear flow is shown schematically in Figure 2:

The normal biconcave shape of the discocyte is transformed into a stomatocyte. These stomatocytes have the same surface area and cell volume as the discocytes from which they originated [78,79,80]. However, altered membrane shear and bending modulus were observed during the erythrocyte’s stomatocyte–discocyte shape transformations [81], leading to the membrane stiffening, caused by structural changes in the actin cortex and lipid bilayer [82]. A stiffer cell membrane caused by the mobility of cholesterol and phosphatidylserine and the rearrangement of the actin cortex reduces the activity of mechanosensitive Piezo1 channels [58]. Piezo1 activation is responsible for the influx of calcium. Calcium activates calpain, a protease that cleaves spectrin and other cytoskeletal proteins, leading to cytoskeletal weakening and increased deformability [67]. On the other hand, it has been demonstrated that calcium influx results in an increase in the amount of membrane-bound hemoglobin [32], which, in turn, leads to a decrease in the erythrocyte’s deformability [83].

While this scenario (i.e., scenario 1) is closely connected with an increase in osmotic pressure, scenario 2 considers the possible response of discocytes under isotonic conditions in the presence of albumin under higher shear rates.

### 2.2. Scenario 2 of Cell Response

This scenario analyses the electrostatic interactions between the erythrocyte membrane and the surrounding solution under isotonic conditions. The surface of erythrocytes is negatively charged primarily due to sialic acid residues on membrane glycoproteins and glycolipids [84,85]. The zeta potential of the membrane in young erythrocytes is around −15 mV, whereas older cells exhibit a zeta potential of −12 mV [20]. Younger cells are more stable than older ones [86]. As negative charge plays a crucial role in maintaining the stability of erythrocytes by ensuring repulsion among cells, it follows that the zeta potential is correlated with the mechanical stability of the erythrocyte membrane [87]. A decrease in zeta potential has a feedback impact on (i) the rearrangement of lipids and, consequently, on the fluidity of the bilayer, (ii) the coupling between the bilayer and actin cortex, (iii) the clustering of band 3 proteins, and (iv) the activity of Piezo1 channels [38]. Silva et al. [86] demonstrated that during storage, the stiffness of red cells increases simultaneously with a decrease in their zeta potential. The altered rearrangement of lipids is one of the key factors responsible for reducing the amount of stomatin. Changes in membrane structure result in increased stiffness and fragility of the cell [61].

The zeta potential represents a measure of the electrical charge of the two sublayers that envelop erythrocytes. The inner sublayer (i.e., the Stern layer) is primarily composed of small cations, such as sodium (Na^+^) and potassium (K^+^) ions, which interact electrostatically with the negatively charged groups present on the cell membrane’s glycoproteins and glycolipids [19]. These interactions help maintain the structural integrity and electrochemical balance of the erythrocyte membrane [18]. The outer sublayer consists of adsorbed plasma proteins, notably negatively charged albumin. Its net negative charge facilitates electrostatic interactions with the positively charged Stern sublayer. These charged sublayers reduce the friction of cells along blood vessels and amortize cell–cell collisions during blood flow [88]. The presence of albumin in the outer sublayer significantly stabilizes erythrocytes at lower and moderate shear rates.

**Figure 3:**
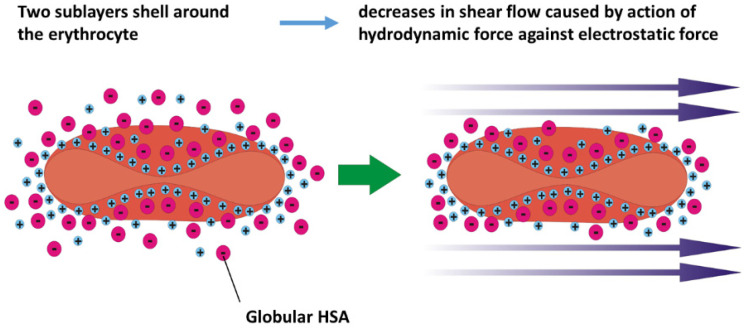
Schematic presentation of the decrease in thickness of the two-sublayer shell around the erythrocyte in shear flow. The thickness of the sublayers, along with the zeta potential, decreases as the shear rate increases within a higher shear rate regime, especially when the hydrodynamic force exceeds the electrostatic force.

However, the thickness of the sublayers, together with the zeta potential, decreases with an increase in shear rate within a higher shear rate regime when the hydrodynamic force is higher than the electrostatic force [21]. A schematic presentation of the decrease in the zeta potential with shear rate is shown in Figure 3:

It follows that the distribution of ions represents the result of a balance between electrostatic force and hydrodynamic force. The hydrodynamic force increases with shear rate, whereas the electrostatic force is influenced by the membrane’s charge and the distribution of ions in its surroundings.

## 3. Conclusions

Erythrocytes’ mechanical stability and deformability are crucial for maintaining their functionality in the bloodstream. Cells encounter significant shear stress, reaching up to 10 Pa levels under physiological conditions as they traverse capillaries. In pathological conditions, this shear stress can be significantly higher. Human serum albumin (HSA) plays an essential role in stabilizing erythrocytes by influencing their shape, membrane integrity, and resistance to hemolysis. Although the impact of albumin on mechanical stability has been extensively studied, the underlying mechanisms by which albumin affects red blood cells remain unclear. Some authors have pointed out that HSA enhances the mechanical stability of erythrocytes, while others argue for the opposite scenario; therefore, a deeper theoretical analysis is crucially needed. When considering this phenomenon, it is essential to note that for healthy cells, hemolysis becomes possible only under flow conditions at high shear stress levels. Under these conditions, the unfolding of the albumin molecule becomes feasible. For this reason, here we have explored the potential influence of albumin unfolding on the mechanical stability of cells.

This review highlights the impact of blood viscosity and anisotropy on the interactions between albumin and the erythrocyte membrane, which becomes more pronounced at higher shear rates, subsequently affecting the mechanical stability of erythrocytes. We base our conclusions/suggestions on a combination of experimental and theoretical analysis, as follows:

- Two possible scenarios of cell response under higher shear stress in the presence of HSA were considered, depending on the ability of albumin to unfold in shear flow. One scenario discusses the electrostatic and hydrodynamic interactions between albumin in its native state and the erythrocyte membrane. The other scenario describes the consequences of hydrophobic interactions between unfolded albumin and the RBC membrane.
- Electrostatic interactions between albumin in its native state and the RBC membrane under isotonic conditions influence the zeta potential of the membrane and may lead to mechanical stabilization of membranes. An increase in shear rate during the flow of erythrocytes through capillaries causes an increase in hydrodynamic interactions between various ions and the membrane, resulting in a decrease in zeta potential. This decrease in zeta potential can destabilize erythrocytes.
- The ability of albumin to unfold under higher shear rates depends on the anisotropic viscoelasticity of blood. Shear flow does not have the potential to directly induce the unfolding of albumin. However, the shear flow of complex anisotropic fluids, such as blood, causes the generation of extensional flow, quantified by the first normal stress difference, which can become significant at higher shear rates. Extensional flow can lead to the partial unfolding of albumin.
- The unfolding of albumin results in (i) an increase in osmotic stress and (ii) intensive hydrophobic interactions between albumin and the membrane of erythrocytes. These interactions can lead to the transition of discocytes into stomatocytes, a cell form that is smaller, stiffer, and more fragile.
- Stiffening of the membrane of erythrocytes depends on (i) the viscoelasticity of the bilayer and actin cortex and (ii) the coupling between them. The stiffness depends on the rearrangement of band 3 in response to membrane fluctuations induced by shear flow and the intracellular concentration of calcium.

In summary, we suggest a distinction between the influences of albumin on the mechanical stability of erythrocytes in its folded and unfolded forms. In the first state (a folded molecule), the presence of albumin stabilizes the cell, while in the second, it destabilizes it. We also propose two scenarios in which unfolded albumin molecules in a red cell suspension can increase their mechanical fragility.

## Figures and Tables

**Table 1:** Divergent effects of albumin on erythrocyte stability.

Role of Albumin	Mechanics of Erythrocyte Membrane	References
Folding	Stabilizing effect caused by electrostatic interactions under wide range of shear rates	[49]
Folding	Stabilizing effect caused by electrostatic interactions under moderate shear rates	[48,50]
Albumin-induced	Discocyte-to-stomatocyte transition caused by a change in the membrane stiffness and bending modulus of the bilayer	[11,12]
Albumin-induced	Stiffening of the erythrocyte membrane is pronounced with an increase in albumin concentration, which can enhance the fragility of cells	[13]

## Data Availability

Not applicable.

## Abbreviations

The following abbreviations are used in this manuscript:

HSA	Human serum albumin
BSA	Bovine serum albumin
RBC	Red blood cell
OUW	Osmotically unresponsive water
PS	Phosphatidylserine
